# Child Psychiatric Patients Exposed to Intimate Partner Violence and/or Abuse: The Impact of Double Exposure

**DOI:** 10.1177/0886260520978186

**Published:** 2020-12-06

**Authors:** Ole Hultmann, Anders G. Broberg, Ulf Axberg

**Affiliations:** 1 University of Gothenburg, Sweden; 2 VID Specialized University, Oslo, Norway

**Keywords:** abuse, children exposed to domestic violence, domestic violence, mental health and violence

## Abstract

Children’s exposure to intimate partner violence (IPV) and child abuse (CA) is strongly related to later psychological problems. Few studies exist on patients in child and adolescent mental health services (CAMHS) who have been singly or doubly exposed to IPV and/or CA. The overall aim of the current study was to compare self-reported psychiatric symptoms, post-trauma impact, and post-traumatic stress disorder (PTSD) diagnoses among CAMHS patients who had been singly or doubly exposed or had reported no family violence (NoFV). We expected to find more severe symptoms in both singly and doubly exposed patients than in the NoFV group and that double exposure was associated with more severe symptoms than single exposure (to IPV or CA). Finally, we expected to find that higher frequencies of exposure to IPV or CA were related to more psychiatric symptoms, greater post-trauma impact, and a higher likelihood of PTSD diagnosis. We compared psychiatric symptoms, post-trauma impact, and PTSD diagnosis in 578 patients aged 9–17 years with NoFV (n = 287), single exposure (n = 162), and double exposure (n = 129). The influence of gender, age, age of onset, frequency, and traumatic interpersonal events outside the family (IPE) were regressed on psychiatric symptoms, post-trauma impact, and PTSD diagnoses. Patients with double exposure had more severe symptoms than the NoFV group, and patients with single exposure had more trauma-related symptoms than the NoFV group. Double exposure was associated with more severe symptoms than single exposure, and frequency (of exposure to IPV and/or CA) and IPE influenced trauma symptoms and psychiatric symptoms, respectively. Exposure to more than one type of violence is associated with more severe symptoms, but other factors such as frequency of violent acts and IPE are important factors to focus on in future studies and clinical assessment.

The prevalence of exposure to intimate partner violence (IPV) among patients in child and adolescent mental health services (CAMHS) is at least twice that in the general population (Ford et al., 2011; Hultmann & Broberg, 2016; McDonald et al., 2000; Olaya et al., 2010; Ormhaug et al., 2012). Child abuse (CA) is probably also more common among children in CAMHS, but few studies have documented this (Jouriles et al., 1997; Ormhaug et al., 2012; Van Looveren et al., 2017). Many studies in the general population have investigated whether double exposure (i.e., both IPV and CA) is more detrimental than single exposure (either IPV or CA), but results are mixed. Kernic et al. (2003) found more internalizing and externalizing symptoms in children with double exposure than in those with single exposure, and both a meta-analysis by Wolfe et al. (2003) and a mega-analysis by Sternberg et al. (2006) also showed support for more externalizing and internalizing symptoms among children who were doubly exposed. A meta-analysis by Kitzman et al. (2003), however, did not support these findings. Some more recent original studies found no clear support for more externalizing and internalizing symptoms in those with double exposure than those with single exposure (English et al., 2009; Moylan et al., 2010). Sousa et al. (2011), however, found support for the double exposure hypothesis in a study among adolescents, in which double exposure was linked more strongly than single exposure both to more delinquency and depression and to lower levels secure attachment to caregivers. Another recent original study found that CA, alone or combined with exposure to IPV, was the more detrimental of the two forms of family violence (Maneta et al., 2017).

To summarize, results on whether double exposure is more detrimental than single exposure are mixed. Differences in study design and study sample may explain some of the conflicting findings, but other explanations may also be relevant. Factors such as parents’ mental health problems, alcohol and drug use, criminality (Dixon et al., 2007), and children’s elevated risk of exposure to violence outside the family (Turner et al., 2016) may all play important roles in the dynamic between family violence and children’s symptoms and could cause poorer outcomes doubly rather than singly exposed children.

Children’s age at first exposure may also be important. At least one study found more detrimental outcomes of double exposure in younger, but not older, children (Sternberg et al., 2006). On the other hand, single exposure to either IPV or CA experienced from a young age (age of onset), over many years (chronicity), very often (frequency), or very harshly (severity) may be more detrimental than double exposure.

Some of the conflicting findings in comparisons of the effects of double versus single exposure may be due to studies’ not controlling for potentially confounding factors. None of the recent original studies, for example, included frequencies of violent acts, age at onset of violence, or violence outside the family.

Very few studies (Hultmann & Broberg, 2016; Olaya et al., 2010; Van Looveren et al., 2017) have adressed whether CAMHS patients exposed to either IPV or CA are burdened with more serious or different symptoms than those with no exposure, and to our knowledge differences between CAMHS patients with single or double exposure have not been studied. The dynamics of double and single exposure may differ between clinical and population-based samples since CAMHS patients enter care with high levels of symptoms, some of which can play a part in their exposure to family violence (Cuevas et al., 2009). CAMHS patients may also have a lower level of resilience against the effects of family violence. It is therefore important to study the effects of family violence on CAMHS patients, including whether double exposure is associated with higher levels of psychiatric symptoms than single exposure.

The present study is based on a sample of 578 consecutive patients in a CAMHS outpatient unit. We compare self-reported psychiatric symptoms and clinician-assigned diagnoses of posttraumatic stress disorder (PTSD) in subgroups of patients with no reported family violence (NoFV), single exposure (IPV or CA), and double exposure (IPV and CA). Additional analyses include the impacts of gender, age at onset of exposure to violence, parent born abroad, additional exposure to violent interpersonal events (IPE) outside the family, and frequency of exposure to IPV and/or CA. In a previous study in this CAMHS unit (Hultmann & Broberg, 2016), the relative impact of family violence and/or IPE was studied in a subsample (*n* = 305). The limited sample size, however, did not allow calculations based on subgroups of patients exposed to different forms of family violence.

## Violence Exposure and Trauma Symptoms

A meta-analysis of studies on a mixture of children from shelters, community and school settings, and clinical settings found a strong association between trauma symptoms and exposure to IPV (Evans et al., 2008). The studies included in the analysis were small, however, lacked homogeneity, and did not control for possible concurrent CA. A study of IPV-exposed 6-to 9-year-old children enrolled in a community-based intervention in the Netherlands found that children with additional exposure to CA showed more posttraumatic symptoms than those without (Telman et al., 2016). In a sample of children referred for suspected CA, both single and double exposure were associated with posttrauma symptoms (Showalter et al., 2019), and in a small study, Kilpatrick & Williams (1998) found that both exposure to IPV and CA were related to diagnoses of PTSD. Prior studies thus make it probable that CAMHS patients exposed to IPV or CA are at risk of developing PTSD, but no such study has been conducted in relation to single versus double exposure.

## Frequency, Duration, and Severity

Studies on the frequency, duration (chronicity), and severity of exposure to IPV and CA are few, and their findings are mixed. Kilpatrick & Williams (1998) found that the frequency of IPV incidents that children witnessed was not associated with their trauma symptoms. Lamers-Winkelman et al. (2012) found no associations between the duration and severity of IPV and children’s externalizing, internalizing or trauma symptoms, nor did Telman et al. (2016) find associations between severity and duration of exposure and children’s trauma symptoms. In contrast, Kernic et al. (2003) found that duration of exposure to IPV and CA were related to more internalizing and externalizing symptoms. As far as we know, no studies have addressed the influence of frequency and duration of exposure to IPV or CA in CAMHS patients.

## Age at First Exposure

Few studies have investigated whether age influences the association between exposure to family violence and psychological symptoms in children. In their mega-analysis, Sternberg et al. (2006) found clear evidence of internalizing problems in doubly exposed children than in those exposed only to IPV for all age groups (4–17 years) and a greater risk of externalizing problems among younger (4–9 years), but not older (10–17 years), children.

## Co-occurrence of Family Violence and Other Forms of Victimization

Other kinds of violence, including violent IPE outside the family, may contribute to the impact of family violence (single or double exposure) on children (Finkelhor et al., 2007a; Turner et al., 2016). In a clinical sample, exposure to IPE outside the family was shown to influence the association between family violence and psychological symptoms (Hultmann & Broberg, 2016). Although they took different risk factors into account, the studies of double exposure by Moylan et al. (2010) and Sousa et al. (2011) did not consider the possible effect of violence outside the family.

## Aims

The main aim of the current study was to compare self-reported psychiatric symptoms, posttraumatic impact, and clinician-assigned PTSD diagnoses between CAMHS patients who had been singly or doubly exposed and patients who reported NoFV. Based on the results of prior studies in the general population and in CAMHS patients, our hypotheses were that compared with patients who reported NoFV, both singly and doubly exposed patients would

- report higher levels of psychiatric symptoms and posttraumatic impact, and
- more often be assigned a PTSD diagnosis by clinicians.

We also expected that

- doubly exposed patients would have more psychiatric and posttraumatic symptoms than singly exposed, and would more often be assigned a PTSD diagnosis, and
- more frequent experiences would be related to more psychiatric symptoms, posttraumatic impact, and PTSD diagnoses.

## Method

### Procedure

We restricted the sample to children old enough to self-report exposure to and impact of violence, and we narrowed family violence to those types most easily self-reported at intake: having been subjected to physical or sexual abuse and/or having been exposed to IPV.

Inclusion criteria required that patients (a) attended a general outpatient CAMHS, (b) were 9 to 17 years old, and (c) had reported exposure to IPV and/or CA (physical or sexual). The Lifetime Incidence of Traumatic Events (LITE) questionnaire was used to assess exposure to violence (see Measures). Interpreters were used with a few children who did not speak Swedish.

Data were collected under the supervision of the research team over a three-year period (March, 2011 to March, 2014) by clinicians (psychologists, social workers, and psychiatric nurses) who regularly conducted intake interviews. Children’s self-reported exposure to IPV and CA, perceived negative impact of CA/IPV, and psychiatric symptoms were collected at intake using questionnaires (see Measures). The children’s symptoms were clinically assessed, and psychiatric diagnoses were assigned by the clinicians who administrated the questionnaires. The research team obtained diagnostic information from the charts after the initial assessment. Ethical approval was granted by the regional ethical committee (D.nr 166-11).

### Participants

The outpatient unit is located in a city with approximately half a million inhabitants. The catchment area of the CAMHS unit has almost three times more people on social welfare and with a foreign background than the city as a whole, and more than twice as many unemployed (Statistics and Analysis City Management Office City of Gothenburg, 2020).

Answers on the LITE questionnaire were used in the analyses comparing the double-exposure, single-exposure, and NoFV groups. Of the 578 participating children, 287 (49.7%) reported NoFV, 80 (13.8%) reported CA only, 82 (14.2%) exposure to IPV only, and 129 (22.3%) double exposure (exposure to both IPV and CA).

The sample consisted of 303 girls and 275 boys (mean age 13.49 years, SD 2.45) evenly distributed in each of the double-exposure, single-exposure, and NoFV groups. Of the 578 children, 232 (41.2%) lived with both parents, 257 (44.5%) with one parent, and 83 (14.3%) in an institution. In 355 (61.4%) of the families, at least one parent was born outside Sweden. There was a statistically significant difference among the three groups (χ^2^ = 8.787, *p* = .01) in where parents were born, and the cell that contributed the most to the difference was doubly exposed children with one or more parents born outside Sweden (standardized residual = −1.9).

The selected patients had been referred for neuropsychiatric problems and/or externalizing problems (23.9%, *n* = 134), depression, suicide attempt, or self-mutilation (25.8%, *n* = 149), other internalizing problems (16.6%, *n* = 100), child maltreatment, family relational problems, or dysfunction in the family (32.9%, *n* = 190), or unknown causes (missing information; 0.8%, *n* = 5). Most study patients were self-referred (37.4%, *n* = 216) but others had been referred to CAMHS by their school (19.4%, *n* = 112), social services (18.3%, *n* = 106), health care (20.6%, n = 119), or somewhere/someone else (4.3%, n = 25).

*Attrition*. A total of 830 children aged 9 to 17 years visited the unit during data collection. Of those, 252 (30%) were not presented with or did not answer the LITE questionnaire. Clinician’s reasons for not asking routine questions in CAMHS about violence in the family have been documented in another study (Hultmann et al., 2014).

### Measures

*Psychiatric diagnoses*. PTSD diagnoses according to DSM-IV criteria were collected through patient charts. For those who had two or more diagnoses, PTSD was chosen over all others.

*LITE.* The LITE questionnaire (Greenwald & Rubin, 1999) was used to obtain children’s self-reported exposure to violence because it was the only psychometrically tested screening instrument for CA in Sweden (Nilsson et al., 2010). Children were seen in private as part of the regular intake procedure and asked by the clinician to fill in a questionnaire about “bad things that can happen to children.” Each item asked whether a type of event had ever happened, how many times it had happened, the respondent’s age at the first occurrence, how much it upset the child then, and how upsetting it was still. Items included eight noninterpersonal events: car accident, other accident, inpatient at a hospital, saw someone get hurt, family member hurt or in hospital, family member died, friend sick, hurt, or died, and house fire or natural catastrophe and seven IPE including the four family violence items presented below, parental separation, being threatened, and being robbed. Additional information was obtained about whether physical, emotional, and/or sexual abuse was committed within the family (exposure to IPV or CA) or outside (IPE) or both.

The instrument’s test–retest reliability was found satisfactory in a community-based study in teenagers (Nilsson et al., 2010). IPE on the LITE scale, especially the items on exposure to IPV and physical abuse (Nilsson et al., 2010), have been shown to be related to scores on trauma scales (Greenwald & Rubin, 1999). The validity and reliability of the LITE questionnaire has not been studied in clinical populations, nor to our knowledge have the measures of frequency, impact of violence, or age at onset. One item in the LITE questionnaire was used to capture witnessing IPV: parents (or adults) broke things in the home or hurt each other. Three items were used to capture CA: (a) having been hit, whipped, beaten, or hurt by someone at home, (b) having been tied up or locked up in a small space at home, and (c) having been sexually abused by a family member. If one of these items was answered positively, CA was registered as having occurred. The children then rated how distressed they were by the events as “not at all,” “a little,” or “a lot.”

*IPE outside the family.* The CA questions on LITE were rephrased, asking whether the child had been subjected to any of these three events by someone outside the family. If one of the three IPE items was answered positively, IPE was registered to have occurred.

*LITE age.* Age at first exposure to IPV or experience of CA was recorded. The lowest age reported for any of the three abuse questions was registered.

*LITE frequency.* Children were asked how many times they had been abused or exposed to IPV. Many children (*n* = 104) did not respond with a number, but used adjectival phrases such as “many times or often,” or “occasionally.” Responses were categorized on a 5-point Likert scale: 0 = 0 times or “never,” 1 = once or twice, 2 = 3 to 6 times or “occasionally,” 3 = 7 to 10 times or “several times,” 4 = 11 times or “many times” (e.g., “as long as I remember,” “every Friday since I was seven years old,” “many, many years”). The number of times children reported witnessing IPV and the highest frequency on any of the two CA questions were counted separately.

*LITE impact.* Children were asked how much the event had upset them (“not at all,” “a little,” “a lot”) at the time of the posttrauma interview. The highest reported impact score on any of the items of family violence post trauma were recorded separately to document posttraumatic impact and subsequently collapsed into one outcome measure. The same procedure was used to take a composite measure of the posttraumatic impact of the remaining items.

*The Strengths and Difficulties Questionnaire (SDQ).* This was used to measure psychiatric symptoms among teenage patients aged 13 to 17 years (*n* = 395). The SDQ symptoms scales rank emotional, conduct, hyperactivity/inattention, and peer problems. Each of these scales has five items scored on 3-point scales ranging from 0 (“never”) to 2 (“often”). The sum of the 20 items on the four scales yields a total score for psychiatric problems ranging from 0 to 40 points. The internal consistency (Cronbach’s alpha) was 0.70 for the emotional scale, 0.59 for the conduct subscale, 0.73 for the hyperactivity scale, 0.67 for the peer relation scale, and 0.76 for the SDQ-total scale.

A total of 97 of the teenagers did not answer the SDQ. There was no statistically significant difference in exposure to violence (IPV or CA) between responders and non-responders to the SDQ (χ2 [1,395] = 2.055, *p* < .152).

### Data Analysis

Since we collapsed participants exposed to CA only or IPV only into one group with single exposure, we controlled for any statistically significant differences between the CA and IPV groups in regard to self-reported psychiatric symptoms, posttraumatic impact, and clinician-assigned diagnoses. No such differences were found, and the collapsed single exposure group was used in the following analysis.

In the first step, associations between exposure to family violence and psychiatric symptoms (SDQ & LITE posttrauma) were examined in a linear regression analysis, controlling for age and gender, IPE outside the family, and (because of the significant distribution in each of the groups) at least one parent born abroad. We created a dummy variable for the three exposure groups and used the NoFV group as a reference. In a second step, logistic regression was used to examine associations between each of the exposure groups and assignments of a diagnosis of PTSD.

To explore differences between singly and doubly exposed participants, another linear regression analysis was run for the three groups in which the double-exposure group was used as a reference. A new logistic regression was conducted to explore potential differences in PTSD assignment in the single- and double-exposure groups.

Next, we examined in a linear regression the associations between experiences of family violence (frequency of CA and/or exposure to IPV), self-reported psychiatric symptoms, and LITE posttraumatic impact, controlling for age, gender, age of first experience, IPE, and at least one parent born abroad. To further test the hypothesis of different outcomes between single and double exposure, we added interaction effects of IPV and CA to the model. To handle potential problems of multicollinearity, we used centered variables of exposure to IPV and CA in the analyses of interaction effects (Howell, 2012). Finally, the association between experience of violence and being assigned a PTSD diagnosis was examined in a logistic regression.

## Results

### Single and Double Exposure Versus No Family Violence

In line with our first hypothesis, doubly exposed patients reported statistically significantly higher levels of general psychiatric symptoms and posttraumatic impact than the NoFV group (Table 1). The double-exposure group also had statistically significantly higher levels of conduct problems and hyperactivity/inattention problems, but not of emotional or peer problems. In the single-exposure group, the hypothesis was confirmed only for posttraumatic impact.

**Table 1.** Linear Regression Models Comparing Dummy Variables of Exposure Groups.

	**Double Exposure vs. No Fam. Violence**			**Single Exposure vs.** **No Fam. violence**			**Single Exposure vs**. **Double Exposure**		
**Measure**	B	SE	*p*	B	SE	*p*	B	SE	*p*
SDQ emotional problems	.30	.38	.43	−.23	.33	.50	−.52	.41	.21
SDQ conduct problems	.98	.29	.00	.28	.25	.26	−.70	.31	.02
SDQ hyperactivity-inattention problems	.85	.40	.04	.14	.35	.69	−.71	.44	.10
SDQ peer problems	−.09	.33	.81	.25	.28	.39	.33	.35	.36
SDQ total problems	2.05	.90	.02	.44	.78	.58	−1.61	.97	.10
LITE post-traumatic impact	.71	.09	.00	.36	.09	.00	−.35	.10	.00

*Note.* Controlled for age and gender and IPE outside the family and birth country of parents.

When data were rerun using the double-exposure group as a reference, the single-exposure group reported statistically significantly lower mean levels of conduct problems and posttraumatic impact than the double-exposure group (Table 1), but not of emotional, peer, or hyperactivity/inattention problems or general psychiatric symptoms.

Members of both exposed groups were more often diagnosed with PTSD than those in the NoFV group (single: B = .81, SE = .37, Exp [B] = 2.27, *p* = .03; double: B = 2.07, SE = .34, Exp [B] = 7.91, *p* < .00). In addition, those in the single-exposure group were statistically significantly less often assigned a PTSD diagnosis than the doubly exposed (B = −1.25, SE = .33, Exp [B] = .27, *p* < .00).

### Frequency of Experiences of IPV or CA

Our hypothesis that higher frequencies of exposure to IPV or CA would be related to more psychiatric symptoms and more posttraumatic impact was confirmed only for posttraumatic impact (Table 2).

**Table 2.** Regression Models of Frequency of Exposure to IPV and CA.

**SDQ-Total**	**B**	**SE**	**β**	***p***	**Lite Post impact**	**B**	**SE**	**β**	***p***
Age	.76	.35	.18	.03	Age	−.02	.02	-.06	.32
Gender	−.67	1.00	−.06	.51	Gender	−.15	.10	−.09	.13
Age at first experience	−.09	.13	−.06	.50	Age at first experience	.03	.02	.14	.05
Parent born abroad	−1,76	.97	−15	.07	Parent born abroad	.02	.10	.01	.84
IPE	2.88	1.03	.23	.01	IPE	.23	.10	.13	.03
Freq. of IPV	.12	.35	.03	.74	Freq. of IPV	.13	.04	.24	.00
Freq. of CA	.49	.35	.12	.16	Freq. of CA	.15	.04	.28	.00
Constant	4.71	5.82		.41	Constant	1.90	.35		.00

*Note.* Controlled for age, gender, age of first experience, parent born abroad, and IPE outside the family.

IPE = traumatic interpersonal events outside the family; Freq. = frequency.

No statistically significant effect was found when the interaction of exposure to IPV and CA was added to the model, thus these results are not presented.

Our hypothesis that higher frequencies of exposure to IPV or CA would be related to more assignments of a PTSD diagnosis was confirmed only for IPV (Table 3).

**Table 3.** Logistic Regression Models of Frequency of CA and Exposure to IPV Predicting Diagnoses of PTSD.

	**B**	**SE**	**Wald**	***p***	**Exp (B)**
Age	.15	.08	3.80	.05	1.16
Gender	−.11	.36	.10	.76	.90
Age of first experience	−.00	.05	.00	.95	1.00
Parent born abroad	.21	.37	.32	.57	1.23
IPE	.42	.37	1.32	.25	1.52
Freq. IPV	.36	.12	9.19	.00	1.43
Freq. CA	.32	.12	1.06	.30	1.13
Constant	−4.41	1.33	11.07	.00	.01

*Note.* Controlling for age, gender, age of first experience, parent born abroad and IPE outside the family.

Wald = Wald test; Exp = exponent of the log odds; IPE = traumatic interpersonal events outside the family.

No significant changes were found when we added the interaction term to the analysis.

## Discussion

The overall aim of the present study was to compare psychiatric symptoms, posttraumatic impact, and clinician-assigned PTSD diagnoses among groups of patients in CAMHS who had not been exposed to family violence and those who had been singly or doubly exposed. The first hypothesis was that both singly and doubly exposed patients would report higher levels of psychiatric symptoms and posttraumatic impact and more often be assigned a PTSD diagnosis than patients who did report no family violence. We also expected doubly exposed patients to have higher levels of symptoms, post-traumatic impact, and PTSD diagnoses than singly exposed children and more frequent exposures in both exposed groups to be related to more psychiatric symptoms, posttraumatic impact, and PTSD diagnoses.

Our first hypothesis was partly confirmed. Doubly exposed patients had more general psychiatric symptoms, more posttraumatic impact, and a higher rate of PTSD diagnoses than the NoFV group. In the single-exposure group, however, the hypothesis was confirmed only for posttraumatic impact and PTSD diagnoses.

In meta-studies, more externalizing and internalizing symptoms have been documented in children exposed to IPV alone (Evans et al., 2008) or to IPV and/or CA (Kitzman et al., 2003; Sternberg et al., 2006; Wolfe et al., 2003) than in children with no such exposure. Externalizing symptoms (conduct and hyperactivity/inattentive problems) were higher in the double-exposure group in our study than in the NoFV group. Elevated conduct problems in the double-exposure group reflect findings from prior studies (Maneta et al., 2017; Moylan et al., 2010), and youth with double exposure have also been previously found to engage in more antisocial behavior than NoFV groups (Sousa et al., 2011). The hyperactivity subscale of the SDQ describes symptoms characteristic of two clusters of PTSD symptoms: hypervigilance and concentration problems. The self-reported hyperactivity/inattentive symptoms may thus be partly driven by traumatic stress reactions. Another possible explanation is that children with hyperactivity/inattention problems are at greater risk for CA (Stern et al., 2018). Given the frequent co-occurrence of CA and exposure to IPV, this might also be valid for exposure to IPV. It is thus important to investigate the dynamics and causality between family violence and hyperactivity/inattention symptoms, which can work both ways (Fuller-Thomson et al., 2014).

Levels of internalizing problems (emotional problems and peer problems), as defined by Goodman et al. (2010) in the SDQ, were no higher in the double-exposure group than in the NoFV group. Our results deviate, however, from most other studies such as that by Maneta et al. (2017), which found more internalizing problems in exposed children than in nonexposed children. A possible explanation for this difference between our study and others might be that we studied a clinical sample of children who tend as a group already to have elevated emotional problems that could be related to family violence or to other factors.

In line with studies using the Trauma Symptom Checklist for Young Children (TSCYC; Lamers-Winkelman et al., 2012; Showalter et al., 2019; Telman et al., 2016), we found an association between double exposure and posttraumatic reactions. Our measurement of posttraumatic impact, a subset of posttraumatic symptoms measured on the LITE scales, also captures how troublesome the memories of exposure to IPV can be.

We found that both singly and doubly exposed children were more often given a PTSD diagnosis than children with no exposure to IPV or CA. In a sample of mothers and children affected by IPV, Castro et al. (2017) found that children’s exposure to IPV and to CA were the two largest contributors to their developing PTSD. Studies of the relationship between exposure to IPV and/or CA and PTSD diagnosis in a CAMHS context are few, but Olaya et al. (2010) documented an association between exposure to IPV and PTSD diagnoses in children in CAMHS, whereas Van Looveren et al. (2017) found no association between CA and a greater risk of a PTSD diagnosis. Methodological factors that might explain the different results of our study and those of Van Looveren et al. are their restricted sample size, low response rate, and the few additional risk factors among their patients. Another interpretation, however, is that CAMHS patients may be more susceptible to developing PTSD from exposure to IPV than to CA.

### Single Exposure Versus Double Exposure

Our third hypothesis, that double exposure is associated with more psychiatric symptoms, greater posttraumatic impact, and more PTSD diagnoses than single exposure, was partly confirmed. Doubly exposed patients had higher levels of self-reported conduct problems, in line with the study by Sousa et al. (2011), who found more antisocial behavior in the doubly exposed than the singly exposed. Also, Maneta et al. (2017) found more externalizing symptoms in the doubly exposed, while Moylan et al. (2010) did not. Our results regarding conduct problems are thus in agreement with those obtained by Maneta et al. and Sousa et al, but general psychiatric symptoms among the doubly exposed in our study were no more severe than those of the singly exposed.

We found that PTSD diagnoses were more strongly related to double exposure than to single exposure. This result reflects that of Telman et al. (2016), who found that CA in combination with exposure to IPV was associated with more trauma symptoms. We have not found studies comparing double and single exposure in relation to PTSD diagnoses.

### Frequency of Exposure to IPV and/or CA in Relation to Psychiatric Symptoms and PTSD Diagnosis

Frequency of exposure to IPV and/or CA was not correlated with psychiatric symptoms, but was associated with more posttraumatic impact; higher frequency of IPV was also associated with more assigned PTSD diagnoses. These findings are contrary to the studies by Hickman et al. (2013) and Kilpatrick and Williams (1998) of young children exposed to IPV and/or CA, both of which found no associations between frequency of exposure to IPV and posttraumatic reactions. The study by Kilpatrick & Williams was small (*n* = 20), which might explain why frequency, among many other factors, did not show any significant associations with PTSD. The study by Hickman et al. measured lifetime frequency of exposure to IPV and trauma symptoms using TSCYC. The mean age of participants was 5 years. For children at that young age, frequency of violent acts may not be as important as it is for older children.

Duration/chronicity of exposure to violence was not associated with more severe emotional, behavioral, or trauma symptoms (Lamers-Winkelman et al., 2012; Telman et al., 2016). The authors explain the lack of effect of chronicity in these studies by the fact that most participants were exposed to family violence for many years, and there was therefore no measurable difference in that variable.

When we added the interaction effects of IPV and CA in the regression model to test the hypothesis of the difference between single and double exposure, we found no statistically significant interaction effect. This indicates that the different effects of single and double exposure might at least partly reflect more frequent violence in the doubly exposed group, indicating the importance of assessing not only different categories of violence, but also factors such as frequency. In line with Hickman et al. (2013), frequency was not associated with more externalizing or internalizing symptoms (SDQ-total).

### The Importance of Other Adverse Exposures

We controlled for IPE outside the family, and this factor was indeed significant in the regression model for general psychiatric symptoms and posttrauma impact. Other than age, IPE was the only factor that significantly predicted increased general psychiatric symptoms (SDQ-total). IPE also significantly predicted trauma reactions (LITE post-traumatic impact), as did frequency of exposure to IPV and/or CA. This finding is in line with the concept of poly-victimization (Finkelhor et al., 2007a, 2007b; Hickman et al., 2013). IPE outside the family have damaging effects on children’s psychological well-being. Violence inside the family may in fact be a precursor of exposure to violence outside the family. In our previous study in CAMHS we found that patients reported their first exposure to IPV or CA had occurred at a younger age than their first experience of IPE (Hultmann & Broberg, 2016).

The associations we found between IPE and symptoms are consistent with the framework of Finkelhor et al. (2007a) and Turner et al. (2016), who showed that victimization in different domains is central to explaining trauma and other psychological symptoms. A recent study in a high-risk sample of children also found support for the hypothesis that exposure to violence in different domains increases the risk for more trauma symptoms (Voith et al., 2014). None of the self-reported psychiatric symptoms in our study, though, were associated with frequency of exposure to IPV or CA; this finding further indicates the strong relationship between severity of violence and trauma symptoms.

Our study of child psychiatric patients indicates that early onset of exposure to IPV and/or CA may worsen trauma reactions but not general psychiatric symptoms. In the mega-analysis by Sternberg et al. (2006), early age of onset of family violence for 5- to 14-year-olds was not related to more severe internalizing or externalizing problems, which is consistent with both our findings and those of a study of CA in 1- to 9-year-olds (Jaffee & Maikovich-Fong, 2011). However, patients in our study reported more posttraumatic impact if they also reported early onset of exposure to IPV or CA, which highlights the importance of examining timing, as proposed by Lacey and Minnis (2019).

## Limitations

Although the LITE questionnaire has shown good psychometric characteristics, it has some limitations. Our translation of children’s verbal answers on the frequency of exposure to IPV and/or CA could be imprecise. Frequency was not normally distributed on our 5-point scale. Only 2 of the 578 children did not report frequency of violence, but 38% failed to answer the frequency questions numerically. The associations of frequency with the dependent variables might have been influenced by those imprecise answers. This finding raises concern about using children and teenagers in research estimating the amount of violence they have been exposed to.

The study did not address causality. CA is well documented as a strong risk factor for an array of emotional and behavioral symptoms, but few studies have been conducted on CA in a CAMHS context. Psychiatric symptoms may also be a risk factor for exposure to violence (Cuevas et al., 2009). Other factors such as the severity and cessation of family violence may also influence posttraumatic impact, but these factors were not measured in this study.

Issues of diversity were not specifically dealt with in this study. Participants were from a low-income area, and almost two thirds of the children had at least one parent born outside Sweden. Low income and possible war-related trauma in immigrant parents may have influenced child–parent relationships and the subsequent levels of symptoms in the children.

Our concept of IPE was restricted, and we did not include child neglect or parental factors such as psychopathology, alcohol and/or drug abuse, criminal behavior, or arrests. The conduct problems subscale in SDQ had a low reliability (.59), so the results from this subscale should be interpreted with caution.

Despite these limitations, our study contributes knowledge about the influence of frequency of exposure to IPV and CA on trauma-related symptoms in doubly exposed CAMHs patients. We found that this subgroup of patients in CAMHS is at risk for more severe symptoms and diagnoses of PTSD.

## Clinical Implications

Our data indicate that CAMHS patients exposed to either IPV or CA have a greater risk of developing trauma-related symptoms than those with no such exposure. Given that almost 50% of the patients in CAMHS reported exposure to IPV or CA, and that 22% reported double exposure, we recommend that clinicians routinely ask patients and parents about family violence. If children’s exposure to IPV is unveiled, clinicians must also consider the possibility of CA and vice versa. Double exposure is associated with a risk of elevated symptoms, and a thorough investigation about the link between exposure to, and experiences of, violence is necessary. Frequency of exposure to IPV and CA is an important aspect to map during the child psychiatric assessment. Age of onset, severity, duration, and type of violence may also be important factors to investigate in relation to psychiatric symptoms and patients’ perceptions of the impact of the violence. Such an investigation could help clinicians to better understand adverse life experiences in relation to psychiatric symptoms and to plan adequate interventions. We recommend using structured questionnaires in the investigation of children’s exposure to and experience of violence and to rigorously consider important aspects of their experience and its impact. Lastly, when family violence is an issue, it is also necessary for the clinician to talk with children about their possible victimization by violence outside the family.

## References

[bibr1-0886260520978186] Castro M., Alcántara-López M., Martínez A., Fernández V., Sánchez-Meca J., & López-Soler C. (2017). Mother’s IPV, child maltreatment type and the presence of PTSD in children and adolescents. *International Journal of Environmental Research and Public Health* , 14(9). 10.3390/ijerph14091077 PMC5615614 28926979

[bibr2-0886260520978186] Cuevas C. A., Finkelhor D., Ormrod R., & Turner H. (2009). Psychiatric diagnosis as a risk marker for victimization in a national sample of children. *Journal of Interpersonal Violence* , 24(4), 636–652. 10.1177/0886260508317197 18445831

[bibr3-0886260520978186] Dixon L., Hamilton-Giachritsis C., Browne K., & Ostapuik E. (2007). The co-occurrence of child and intimate partner maltreatment in the family: Characteristics of the violent perpetrators. *Journal of Family Violence* , 22(8), 675–689. 10.1007/s10896-007-9115-x

[bibr4-0886260520978186] English D. J., Graham J. C., Newton R. R., Lewis T. L., Thompson R., Kotch J. B., & Weisbart C. (2009). At-risk and maltreated children exposed to intimate partner aggression/violence:What the conflict looks like and its relationship to child outcomes. *Child Maltreatment* , 14(2), 157–171. 10.1177/1077559508326287 18984806

[bibr5-0886260520978186] Evans S. E., Davies C., & DiLillo D. (2008). Exposure to domestic violence: A meta-analysis of child and adolescent outcomes. *Aggression and Violent Behavior* , 13(2), 131–140. 10.1016/j.avb.2008.02.005

[bibr6-0886260520978186] Finkelhor D., Ormrod R. K., & Turner H. A. (2007 a). Poly-victimization: A neglected component in child victimization. *Child Abuse and Neglect* , 31(1), 7–26. 10.1016/j.chiabu.2006.06.008 17224181

[bibr7-0886260520978186] Finkelhor D., Ormrod R. K., & Turner H. A. (2007 b). Polyvictimization and trauma in a national longitudinal cohort. *Development and Psychopathology* , 19(01), 149–166. 10.1017/S0954579407070083 17241488

[bibr8-0886260520978186] Ford J. D., Gagnon K., Connor D. F., & Pearson G. (2011). History of interpersonal violence, abuse, and nonvictimization trauma and severity of psychiatric symptoms among children in outpatient psychiatric treatment. *Journal of Interpersonal Violence* , 26(16), 3316–3337. 10.1177/0886260510393009 21362676

[bibr9-0886260520978186] Fuller-Thomson E., Mehta R., & Valeo A. (2014). Establishing a link between attention deficit disorder/attention deficit hyperactivity disorder and childhood physical abuse. *Journal of Aggression, Maltreatment & Trauma* , 23(2), 188–198. 10.1080/10926771.2014.873510

[bibr10-0886260520978186] Goodman A., Lamping D. L., & Ploubidis G. B. (2010). When to use broader internalising and externalising subscales instead of the hypothesised five subscales on the Strengths and Difficulties Questionnaire (SDQ): Data from british parents, teachers and children. *Journal of Abnormal Child Psychology* , 38(8), 1179–1191. 10.1007/s10802-010-9434-x 20623175

[bibr11-0886260520978186] Greenwald R., & Rubin A. (1999). Assessment of posttraumatic symptoms in children: Development and preliminary validation of parent and child scales. *Research on Social Work Practice* , 9(1), 61–75. 10.1177/104973159900900105

[bibr12-0886260520978186] Hickman L. J., Jaycox L. H., Setodji C. M., Kofner A., Schultz D., Barnes-Proby D., & Harris R. (2013). How much does “how much” matter? Assessing the relationship between children’s lifetime exposure to violence and trauma symptoms, behavior problems, and parenting stress. *Journal of Interpersonal Violence* , 28(6), 1338–1362. 10.1177/0886260512468239 23266994

[bibr13-0886260520978186] Howell D. C. (2012). *Statistical methods for psychology* (8th ed.). Wadsworth Publishing.

[bibr14-0886260520978186] Hultmann O., & Broberg A. (2016). Family violence and other potentially traumatic onterpersonal events among 9- to 17-year-old children attending an outpatient psychiatric clinic. *Journal of Interpersonal Violence* , 31(18), 2958–2986. 10.1177/0886260515584335 25917005

[bibr15-0886260520978186] Hultmann O., Möller J., Ormhaug S. M., & Broberg A. (2014). Asking routinely about intimate partner violence in a child and adolescent psychiatric clinic: A qualitative study. *Journal of Family Violence* , 29(1), 67–78. 10.1007/s10896-013-9554-5

[bibr16-0886260520978186] Jaffee S. R., & Maikovich-Fong A. K. (2011). Effects of chronic maltreatment and maltreatment timing on children’s behavior and cognitive abilities. *Journal of Child Psychology and Psychiatry* , 52(2), 184–194. 10.1111/j.1469-7610.2010.02304.x 20735512 PMC2998571

[bibr17-0886260520978186] Jouriles E. N., Mehta P., McDonald R., & Francis D. J. (1997). Psychometric properties of family members’ reports of parental physical aggression toward clinic-referred children. *Journal of Consulting and Clinical Psychology* , 65(2), 309–318. 10.1037/0022-006X.65.2.309 9086695

[bibr18-0886260520978186] Kernic M. A., Wolf M. E., Holt V. L., McKnight B., Huebner C. E., & Rivara F. P. (2003). Behavioral problems among children whose mothers are abused by an intimate partner. *Child Abuse and Neglect* , 27(11), 1231–1246. 10.1016/j.chiabu.2002.12.001 14637299

[bibr19-0886260520978186] Kilpatrick K. L., & Williams L. M. (1998). Potential mediators of post-traumatic stress disorder in child witnesses to domestic violence. *Child Abuse and Neglect* , 22(4), 319–330. 10.1016/S0145-2134(97)00178-6 9589182

[bibr20-0886260520978186] Kitzman K. M., Gaylord N. K., Holt A. R., & Kenny E. D. (2003). Child witnesses to domestic violence: A meta-analytic review. *Journal of Consulting and Clinical Psychology* , 71(2), 339–352. 10.1037/0022-006X.71.2.339 12699028

[bibr21-0886260520978186] Lacey R. E., & Minnis H. (2019). Practitioner review: Twenty years of research with adverse childhood experience scores – Advantages, disadvantages and applications to practice. *Journal of Child Psychology and Psychiatry* , 61(2), 116–130. 10.1111/jcpp.13135 31609471

[bibr22-0886260520978186] Lamers-Winkelman F., Willemen A. M., & Visser M. (2012). Adverse childhood experiences of referred children exposed to intimate partner violence: Consequences for their wellbeing. *Child Abuse and Neglect* , 36(2), 166–179. 10.1016/j.chiabu.2011.07.006 22280846

[bibr23-0886260520978186] Maneta E. K., White M., & Mezzacappa E. (2017). Parent-child aggression, adult-partner violence, and child outcomes: A prospective, population-based study. *Child Abuse and Neglect* , 68, 1–10. 10.1016/j.chiabu.2017.03.017 28388466

[bibr24-0886260520978186] McDonald R., Jouriles E. N., Norwood W., Shinn W. H., & Ezell E. (2000). Husbands’ marital violence and the adjustment problems of clinic-referred children. *Behavior Therapy* , 31(4), 649–665.

[bibr25-0886260520978186] Moylan C., Herrenkohl T., Sousa C., Tajima E., Herrenkohl R., & Russo M. (2010). The effects of child abuse and exposure to domestic violence on adolescent internalizing and externalizing behavior problems. *Journal of Family Violence* , 25(1), 53–63. 10.1007/s10896-009-9269-9 20495613 PMC2872483

[bibr26-0886260520978186] Nilsson D. K., Gustafsson P. E., & Svedin C. G. (2010). Self-reported potentially traumatic life events and symptoms of post-traumatic stress and dissociation. *Nordic Journal of Psychiatry* , 64(1), 19–26. 10.3109/08039480903264846 19883198

[bibr27-0886260520978186] Olaya B., Ezpeleta L., de la Osa N., Granero R., & Doménech J. M. (2010). Mental health needs of children exposed to intimate partner violence seeking help from mental health services. *Children and Youth Services Review* , 32(7), 1004–1011. 10.1016/j.childyouth.2010.03.028

[bibr28-0886260520978186] Ormhaug S. M., Jensen T. K., Hukkelberg S. S., Holt T., & Egeland K. E. (2012). *Barns traumer; gjemt eller glemt? Kartlegging av traumatiske erfaringer hos barn og unge henvist til BUP* [Children’s trauma. Hidden or forgotten? An inquiry into the traumatic experiences of children and adolescents in child and adolescent mental health care]. *Tidsskrift for Norsk Psykologforening*, 49, 234–240.

[bibr29-0886260520978186] Showalter K., Yoon S., Maguire-Jack K., Wolf K. G., & Letson M. (2019). Are dual and single exposures differently associated with clinical levels of trauma symptoms? Examining physical abuse and witnessing intimate partner violence among young children. *Child & Family Social Work* , 25(2), 439–447. 10.1111/cfs.12700

[bibr30-0886260520978186] Sousa C., Herrenkohl T. I., Moylan C. A., Tajima E. A., Klika J. B., Herrenkohl R. C., & Russo M. J. (2011). Longitudinal study on the effects of child abuse and children’s exposure to domestic violence, parent-child attachments, and antisocial behavior in adolescence. *Journal of Interpersonal Violence* , 26(1), 111–136. 10.1177/0886260510362883 20457846 PMC2921555

[bibr31-0886260520978186] Statistics and Analysis City Management Office City of Gothenburg. (2020). Statistical database - city of Gothenburg. http://statistikdatabas.goteborg.se/pxweb/sv/1.%20Göteborg%20och%20dess%20delområden/

[bibr32-0886260520978186] Stern A., Agnew-Blais J., Danese A., Fisher H. L., Jaffee S. R., Matthews T., Polanczyk G. V., & Arseneault L. (2018). Associations between abuse/neglect and ADHD from childhood to young adulthood: A prospective nationally-representative twin study. *Child Abuse and Neglect* , 81, 274–285. 10.1016/j.chiabu.2018.04.025 29775871 PMC6013278

[bibr33-0886260520978186] Sternberg K. J., Baradaran L. P., Abbott C. B., Lamb M. E., & Guterman E. (2006). Type of violence, age, and gender differences in the effects of family violence on children’s behavior problems: A mega-analysis. *Developmental Review* , 26(1), 89–112. 10.1016/j.dr.2005.12.001

[bibr34-0886260520978186] Telman M. D., Overbeek M. M., de Schipper J. C., Lamers-Winkelman F., Finkenauer C., & Schuengel C. (2016). Family functioning and children’s post-traumatic stress symptoms in a referred sample exposed to interparental violence. *Journal of Family Violence* , 31, 127–136. 10.1007/s10896-015-9769-8 26709333 PMC4688302

[bibr35-0886260520978186] Turner H. A., Shattuck A., Finkelhor D., & Hamby S. (2016). Polyvictimization and youth violence exposure across contexts. *Journal of Adolescent Health* , 58(2), 208–214. 10.1016/j.jadohealth.2015.09.021 26598060

[bibr36-0886260520978186] Van Looveren N., Glazemakers I., Van Grootel L., Fransen E., & Van West D. (2017). Assessment of Physical Child Abuse Risk in Parents with Children Referred to Child and Adolescent Psychiatry. *Child Abuse Review* , 26(6), 411–424. 10.1002/car.2470

[bibr37-0886260520978186] Voith L. A., Gromoske A. N., & Holmes M. R. (2014). Effects of cumulative violence exposure on children’s trauma and depression symptoms: A social ecological examination using fixed effects regression. *Journal of Child & Adolescent Trauma* , 7(4), 207–216. 10.1007/s40653-014-0026-8

[bibr38-0886260520978186] Wolfe D. A., Crooks C. V., Lee V., McIntyre-Smith A., & Jaffe P. G. (2003). The effects of children’s exposure to domestic violence: A meta-analysis and critique. *Clinical Child and Family Psychological Review* , 6(3), 171–187. https://doi.org/1096-4037/03/0900-0171/0 10.1023/a:1024910416164 14620578

